# Using Interactive Patient Engagement Technology in Clinical Practice: A Qualitative Assessment of Nurses’ Perceptions

**DOI:** 10.2196/jmir.5667

**Published:** 2016-11-11

**Authors:** Frances L Patmon, Perry M Gee, Tina L Rylee, Noriann L Readdy

**Affiliations:** ^1^Nursing Research and AnalyticsDignity HealthPhoenix, AZUnited States; ^2^Betty Irene Moore School of NursingUniversity of California, DavisSacramento, CAUnited States; ^3^College of NursingUniversity of UtahSalt Lake City, UTUnited States; ^4^Department of PsychologyCalifornia State UniversitySacramento, CAUnited States; ^5^Patient Care ServicesDignity HealthSan Francisco, CAUnited States

**Keywords:** patient engagement, health information technology, educational technology, patient education, patient experience, primary care nursing, qualitative research

## Abstract

**Background:**

Research has shown patients who are more engaged in their care are likely to have better health outcomes and reduced health care costs. Health care organizations are now focusing their efforts in finding ways to improve patient engagement. At the forefront of this movement are patient engagement technology systems. In this paper, these emerging systems are described as interactive patient engagement technologies (iPET).

**Objective:**

The objective of this descriptive study was to gain an understanding of the perceptions of nurses who are integrating these iPET systems into their daily clinical practice.

**Methods:**

The research team interviewed 38 nurses from 2 California-based hospitals using a focused rapid ethnographic evaluation methodology to gather data.

**Results:**

The study participants reported that using iPET systems may enhance clinical nursing practice. The 4 key findings of iPET were that it (1) is effective for distraction therapy, (2) has functionality that affects both patients and nurses, (3) has implications for clinical practice, and (4) may require additional training to improve usage.

**Conclusions:**

With sufficient training on the iPET system, nurses believed they could use these technologies as an enhancement to their clinical practice. Additionally, nurses perceived these systems served as distraction therapy for patients. Initial findings suggest that iPET is beneficial, but more research is required to examine the usefulness of iPET systems in the inpatient settings.

## Introduction

It has been over a decade since the Institute of Medicine (IOM) first recommended that patients should have an active role in their health care [1]. Additionally, the IOM highly endorses the integration of information technology in this endeavor [1]. Health information technology (HIT) systems have long been touted as the newest intervention aimed at increasing patient engagement with the end result of improving patient outcomes [2]. Nurses have a unique role in that they are at the juncture of new technologies and patient care in the acute care setting. Historically, nurses have intersected with patients with technologies such as electronic health records (EHRs), intravenous pumps, specialty beds, monitoring and safety equipment, and even items as simple as call lights and television. Therefore, nurses play an integral role in identifying ways to improve patient engagement and optimize the potential benefits of new HIT systems. Initial studies about patient-centered HIT systems in the outpatient setting have shown that they have the potential to engage patients, to facilitate communication with their providers, and to encourage participation in their own care [3].

The definition of patient engagement has varied over the years. The Institute for Healthcare Improvement defined it as the “actions that people take for their health and to benefit from care” [4]. A research team in Australia also defined patient engagement as “a co-constructed process and state” [5]. They further describe patient engagement as a process of gradually connecting with each other and/or a therapeutic program, which enables the individual to become an active, committed, and invested collaborator in health care [5]. Furthermore, the Affordable Care Act identified patient engagement as a key piece in health care reform [6].

Patient engagement has been quoted as the new “blockbuster drug” aimed at improving 3 key things—patient experience, patient satisfaction, and patient outcomes—all while improving health care costs [4]. A recent study found that patients who scored low on the Patient Activation Measure (a scale designed to measure one’s knowledge, skills, and confidence in managing their own health needs), were more likely to have greater health care costs compared to those patients with higher activation scores [6]. Moreover, a systematic review found that using information technology (IT) platforms to increase patient engagement could result in positive outcomes [7].

Health care organizations across the United States have enhanced their HIT systems in an effort to engage the patient [8]. Despite the supposed improvement in patient outcomes with these patient engagement technology systems, most systems are not reaching their full potential. A major barrier to IT adoption is user acceptance [9]; the technology acceptance model (TAM) states that user acceptance is highly influenced by the perceived usefulness of the system [10]. Moreover, a study conducted that looked at call-light technology found that once nurses were shown a full demonstration of the technology, nurses were more willing to use these systems to improve their workflow and, ultimately, the technology had a positive impact on patient outcomes [11].

The goal of the iPET systems is to increase patient engagement through technology. A common example is the patient portal, which allows patients to message their physician, make appointments online, or request medication refills. Although limited, early studies have shown the benefits of patient engagement systems in the inpatient setting; a systematic review indicates that these systems can deliver generic and specific patient education, enhance communication between physicians and patients, provide entertainment, and empower patient decision making [2].

**Figure 1:**
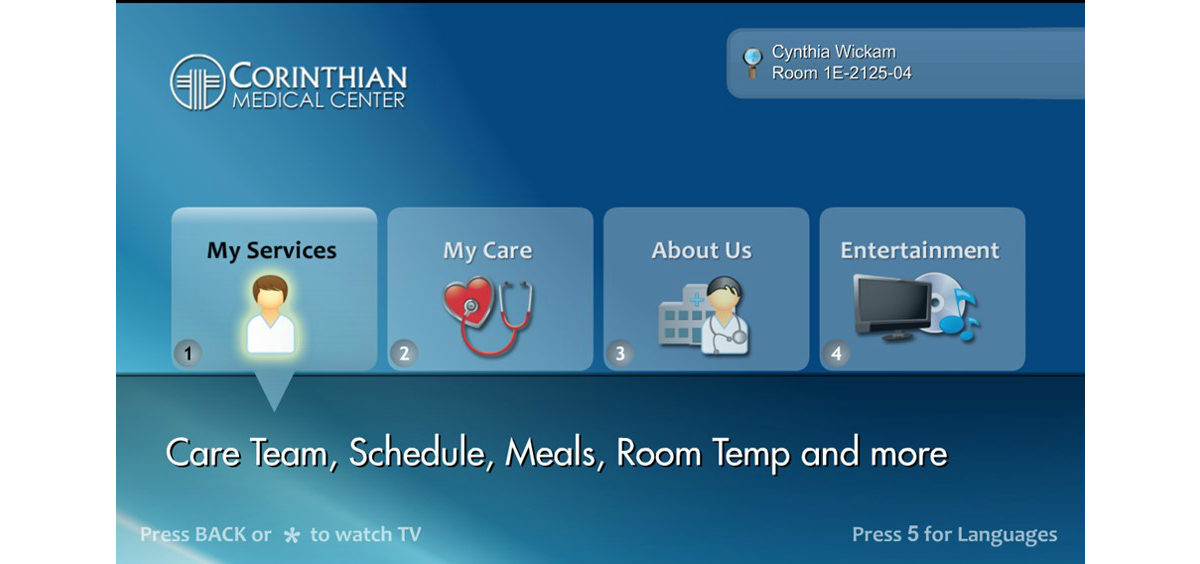
Example of an *interactive patient engagement technology* (iPET) patient menu. Reprinted with permission from SONIFI Health, Inc, Sioux Falls, SD, USA.

**Figure 2:**
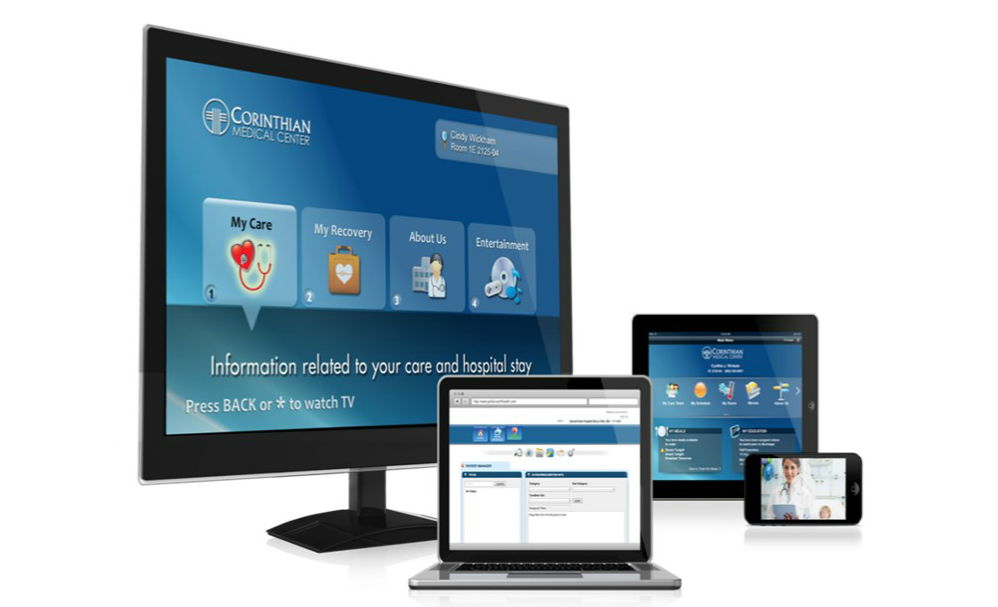
Examples of a variety *interactive patient engagement technology* (iPET) user interface devices. Reprinted with permission from SONIFI Health, Inc, Sioux Falls, SD, USA.

**Figure 3:**
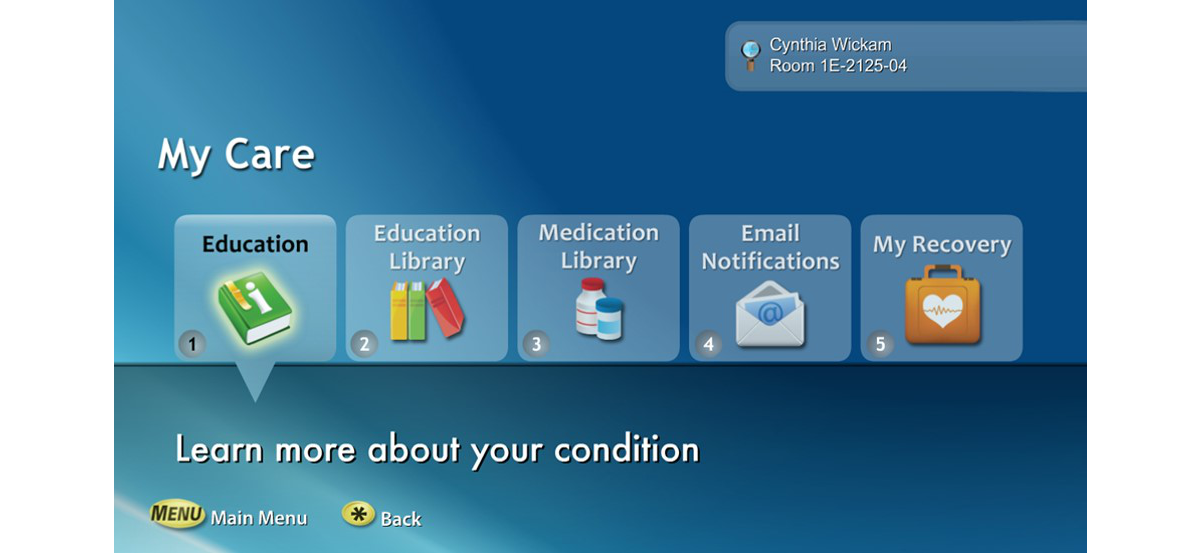
A selection of example *interactive patient engagement technology* (iPET) self-management tools. Reprinted with permission from SONIFI Health, Inc, Sioux Falls, SD, USA.

**Figure 4:**
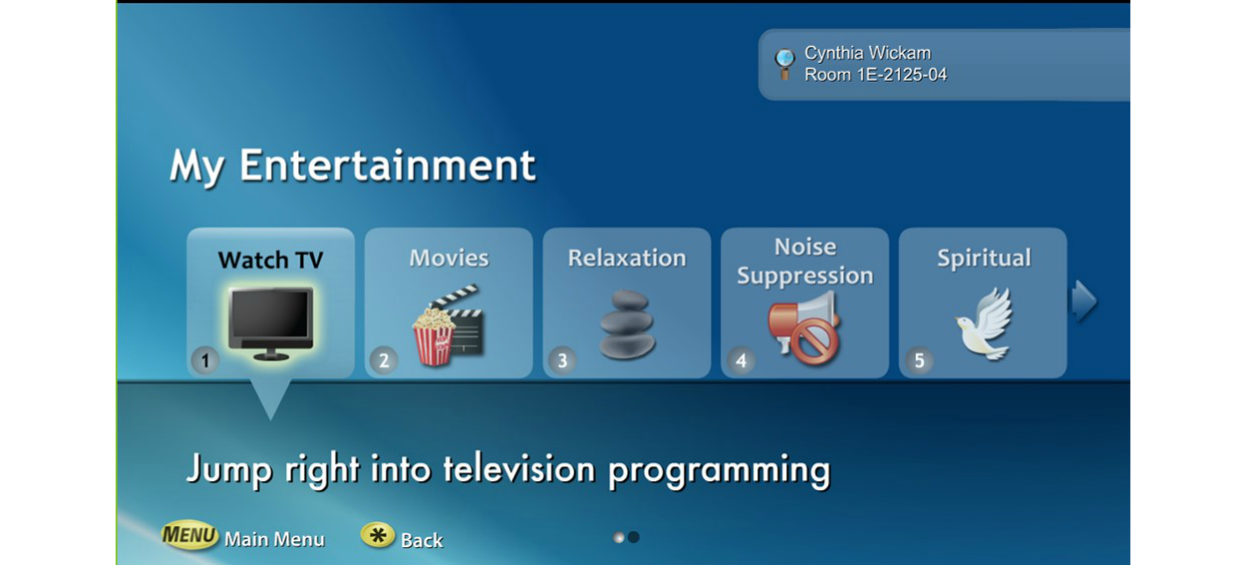
A selection of example distraction therapy options. Reprinted with permission from SONIFI Health, Inc, Sioux Falls, SD, USA.

This study examined nurses’ perception of patient engagement technology systems on their clinical practice in the acute care setting. Our team wanted to identify barriers and promoting factors that affect utilization and usage of patient engagement technology by nurses. We refined the term “patient engagement technology systems” and are introducing a new concept called *interactive patient engagement technology* (iPET). We defined iPET as any electronic system that delivers a bundle of health self-management, communication, education, and distraction services on demand. The iPET systems are used by patients and their families in the inpatient or outpatient setting and are designed to enhance or promote patient engagement in one’s own health care (see Figures 1 and 2). iPET systems may increase patient engagement by providing some or all of the following components: a portal for patient-provider communication, access to the portions of the EHR, patient education on disease processes, diagnostics, and medications (see Figure 3). Additionally iPET systems have the ability to function as distraction therapy by offering spiritual care content, music, movies, white noise, and relaxation techniques (see Figure 4). The interactive component occurs between the nurse, the patient, and the patient’s family and is crucial to the successful adoption of the iPET technology. The delivery of the iPET systems in this study was through iPads in the emergency department (ED) and, in the inpatient setting, where patients had access to the system through the patient’s television in the room. The iPad and television systems in this study contain a variety of entertainment options, spiritual care modules, and patient education materials.

The aim of this study was to examine nurses’ perceptions of patient engagement technology systems during their clinical practice in the acute care setting.

## Methods

Due to the emerging nature of using iPET in the clinical setting and the paucity of supporting evidence in previous literature, an ethnographic qualitative approach was chosen for the initial inquiry [12]. Because of clinical responsibilities and business requirements in the patient care units, access to research participants was limited. The nurses reported they did not have time to participate in interviews and found it difficult to participate in research during the work shift. Furthermore, keeping nurses after the end of the work shift or bringing them in on an off day was not an option due to the financial and collective bargaining contract constraints. Because of these methodological challenges, the research team looked to use a nontraditional, qualitative nursing methodology: focused or rapid ethnography. Moreover, our research team has labeled our unique method as focused rapid ethnographic evaluation (FREE). The FREE method shares many common features of traditional rapid or focused ethnography as described in the literature [13-17], with the exception of our team’s extensive use of field notes in lieu of digital recordings. FREE is especially appropriate for situations where human-computer interactions occur, and where organizations are appraising emerging technologies in the work setting [14,17,18].

### Participants

The authors conducted the study at 2 community hospitals in California. The researchers recruited 38 participants from both hospitals. Purposive sampling was used to include registered nurses who use the iPET as part of their daily practice [19]. To allow for a variation in perspectives on interactive technology and identify key informants, the researchers interviewed nurses who were currently practicing as well as those that fulfilled leadership roles. In an urban hospital in Southern California, the research team interviewed nurses in 2 different departments. In the ED, the researchers interviewed 10 participants with a range of experience from 2 to 40 years: 8 females and 2 males. Additionally, the research team observed 10 participants in the medical-surgical department, with a range of experience from 2 to 25 years: 8 females and 2 males. In an urban hospital in Northern California, the researchers interviewed 2 departments: ED and the family birth center. In the ED, researchers interviewed 10 participants with a range of experience of 5 to 25 years: 7 females and 3 males. Furthermore, in the family birth center, the researchers interviewed 8 female participants with a range of experience between 15 and 30 years.

### Procedure

The research team conducted an initial review of the associated literature and applicable theories. Research that used TAM in the health care setting has consistently shown that clinicians’ perception on the ease of practice and the usefulness of health information technologies determines future intentions and adoption of these systems [10,20]. Influenced by the current literature and the TAM resources, our research team developed a strategy for the project.

As recommended in the literature, prior to starting the participant interviews and observations, key individuals familiar with the newly implemented iPET system were contacted and interviewed by phone and in person [18,21]. These individuals advised the research team to the appropriate departments and suggested a strategy to observe and interview participants in a time and location for optimal data gathering. Based on the initial discussions, the research team developed a semistructured interview guide, a systematic approach to record field notes, identified areas for observation, and scheduled interviews. The final preparation was on the day before beginning the study; members of the research team toured the facility and units to become familiar with the layout and to meet managers, team leaders, and some of the potential participants.

Observations began during the first visit to the departments and continued to the final day of the project. During the course of the study, at least 2 research team members were present for all interviews. Additionally, researcher observations that occurred during the interview process—impressions of the setting, body language, appearance of the participant, use of the iPET system, and other findings—were documented as field notes [22]. The researchers observed the nursing workflow in the individual departments, in patient care areas, nursing stations, hallways, supply and utility rooms, and break rooms. Initially, there was an attempt to have nurses “drop by” the break room for formal and private interviews. However, due to the work-related requirements of the units and patient needs, the nurses spent most of the day in the clinical areas. Some interviews occurred in the quiet break room; however, most occurred at the nurses’ stations located in the hall, near the medication cart, in empty patient rooms, in offices, and other locations where the nurse and interviewers could talk.

Interviews lasted anywhere from 10 minutes to 1 hour. A nurse had to cut one of the interviews short due to a “code-blue” emergency in their department. During all interviews and observations, both the researchers took extensive hand- written field notes in journals. When time allowed or at the end of each interview, the research team compared notes and made any necessary adjustments to the semistructured interview question prompts. The researchers entered empty patient rooms, observed demonstrations of the technologies by the nursing staff, and explored the iPET systems. Finally, the data collection process stopped when “saturation” was obtained or no new data or findings were noted or observed [23].

Data analysis began during the first observations and continued throughout the study. At the end of each day, the researchers compared field notes and began discussing emerging findings and areas that needed further exploration and initial thoughts on themes. Three members of the research team met to organize all data and field notes after data collection was complete and the researchers reached data saturation. The authors combined both observed and interview data, then looked for patterns in the data, and began initial coding. The research team developed a codebook to identify and define broad categories from the data, additionally creating subcategories as they emerged. The authors frequently compared their reasoning for coding specific data in a specific manner and worked as a team to come to consensus. Subsequently, the researchers uploaded the data to MAXQDA version 11 (VERBI GmbH software, Berlin, Germany) qualitative analysis software where the statements were organized and systematically indexed to facilitate categorization. When the analysis was nearly complete, a central theme was identified; the individual codes were defined, resorted, categorized, recategorized; and 4 major findings were established [24]. The 4 findings with subfindings were identified and a presentation was developed to discuss the authors’ overall impression. When analysis was complete, the presentation was formally shared with key participants (unit managers and nursing team leads), and the findings were verified and confirmed as accurate in a process known as *member checking* [25] *.*

The researchers consulted the Institution Review Board at Dignity Health, Sacramento, CA, for approval prior to beginning this study. The researchers provided an explanation of the purpose of the study and the research methods to the nurses before the start of the interviews. Additionally, the researchers informed each participant that observations and data collected was strictly confidential, and that the authors would not identify any individual participant throughout the study. Each participant gave verbal consent, and the researchers told the nurses they could end the interview and withdraw any data contributed to the study, at any time in the process.

Throughout the entire research process, the research team practiced *reflexivity*, which is the process of identifying one’s beliefs and biases related to the research [15]. Since the researcher is the data-gathering instrument in the FREE methodology, our team first shared any preconceptions with one another, questioned each other when unsure about any aspect of the process, and were transparent with each other through the entire data gathering and analysis process to assure as much rigor in the research as possible.

## Results

Overall, the study participants perceived that the use of iPET systems had great potential to enhance their clinical practice. Through data analysis, the 4 key findings or themes identified were (1) effective for distraction therapy, (2) functionality affects both patients and nurses, (3) there are implications for clinical practice, and (4) training may improve usage.

### iPET Is Effective for Distraction Therapy

One of the most powerful uses of iPET was for distraction. The authors categorized distraction into 2 areas: active and passive. Active distraction promotes the involvement of the patient during a procedure, such as games that require participation. In contrast, passive distraction therapy is much less involved, such as listening to music and/or watching television [26]. The researchers found that iPET, along with the associated entertainment, were quite helpful for distraction, especially with patients who were waiting or holding for long periods in the ED. Several of the nurses stated how helpful iPET was for distraction in the ED. One nurse mentioned, “The only problem is when it is time to move the patient to another department; they want to take the iPad with them.” Another ED nurse said, “My patients seem happier and, frankly, I am answering fewer call lights since using the system.” Similarly, several of the nurses revealed that patients seemed to be on their call lights less while waiting in the ED. “The system is very useful for my hold patients in the ED,” said one nurse whose comments were reflected in several other nurses’ responses. Conversely, the tablet computers did not have access to live televisions, which was one problem noted by several nurses in the ED: “Our patients wanted to watch the football game.”

The iPET was especially useful for distraction for children and patients with various psychiatric conditions: “We have had more than one person with mental health issues where the iPads were very helpful in keeping them calm while waiting in the ED.” Nurses also said that the iPET system was helpful as a distraction for some visitors who were waiting with patients in the rooms. In the medical-surgical units, nurses stated that turning on the entertainment or “white noise” portions of the iPET system helped “bedridden patients pass the time.” Furthermore, whether patients used music, white noise, or movies, one of the most useful reasons for implementing the iPET system was for patient distraction and entertainment.

### iPET Functionality Affects Patients and Nurses

Because of the uniqueness of the iPET implementation, functionality of the system appeared to be a common finding among the nurses interviewed. Specifically, the security of the iPad tablet computers used for iPET in the ED was one common finding. Nurses worried about patients “stealing” the iPads; indeed, 2 disappeared early in the implementation. These thefts prompted a change in policy toward the implementation of locking support arms for the iPads in the ED. The locking arms did seem to thwart the concerns over theft, but some ED nurses worried that the patients could use the arms “as weapons” by dismantling them. Additionally, nurses were concerned that the iPad thefts would fall under their liability. For example, one nurse stated, “If I sign out the iPad to a patient and then my shift ends, I won’t be present to sign it back in.” Several nurses reported iPad theft concerns, and the agencies involved in the research were actively working to alleviate those fears and develop a sound policy to assure future success.

The authors identified a variety of technical and implementation issues. One significant issue identified was that the implementations of the systems were dissimilar at the different hospitals. Some departments had a full complement of movies and music offered, whereas others only had select options. At an urban facility in Southern California, one nurse stated, “Many of the patients in our emergency department are from the rap culture, and there is no rap music on this system for them to listen to.” In addition, others reported a limited offering of children’s videos. Overall, nurses recommend customizable entertainment offerings to reflect the local patient population.

Due to the lack of fully implemented and integrated iPET systems, the nurses had several questions about its full functionality, including educational offerings. Ideally, a patient would receive educational materials, that their clinician ordered, on the iPET system, and once the patient viewed the material, the iPET system would update the patient’s EHR. In the units where this functionality was fully implemented, the nurses were very impressed with the how the system could be used for patient education. One experienced labor and delivery nurse stated, “I just order the package of patient education videos, then the patient and family view the videos, and then my job is to facilitate the patient education using a teach-back methodology.” Other nurses mentioned, “I never could cover all the material delivered in the [patient-specific] educational videos; the system is so helpful.” During the course of the interviews, several of the nurses revealed specific videos they would like to see added to the implemented iPET system. For example, more than one medical-surgical nurse stated that videos discussing peripherally inserted percutaneous intravenous for patients transferring home would be helpful. Furthermore, the research team and unit managers will submit suggested education video requests to the vendor.

### iPET Has Implications for Clinical Practice

According to the nurses interviewed, their patients really liked and appreciated the iPET system. The nurses reported that the systems were intuitive for patients to use and they were easily able to help patients who needed instruction using the technology. Nurses used the iPET system to help calm and distract agitated psychiatric patients, patients who were autistic, confused and lonely children, and older adults. Again, the nurses found the systems useful for patients who were “holding” and waiting for long periods or needed distraction for a variety of reasons. One ED nurse stated, “The system helps me calm psychiatric patients,” and several others claim purposely using the system in the same manner. Many of the nurses, specifically on the medical-surgical unit, stated that patients seemed to appreciate the “white noise” feature of the system to help the patient rest and to drown out some of the unit noise.

Although most nurses reported they used the iPET system for distraction, several nurses emphasized that the patient education videos about diseases and medication would help with patient teaching. One particular nurse stated that she incorporated an introduction of the system as part of her initial patient assessment; during this assessment, she encouraged her patients to review medications and disease information specific to them as a starting ground for patient teaching. This nurse reported that after watching the videos, the communication was enhanced because patients had preliminary baseline teaching, which allowed for more interactive communication.

In addition to the education materials mentioned earlier, nurses can use the system to support patient’s spiritual needs. Most major religions have content in the system, including religious texts, teachings, songs or hymns, and mindfulness techniques. Nurses reported encouraging patients to use the spiritual care aspects of the system when desired. Because the iPET system implementation was so new, nurses expressed the desire to have more time to explore and use the system with patients. Overall, many nurses reported that the iPET system “made their job easier.”

### iPET Training May Improve Usage

Because the iPET system is so closely related to familiar tablet computer (iPad) and television technologies, those implementing the system, and the nurses themselves, tended to overlook training needs. Furthermore, the iPET system implementation was so new, nurses wished they had more time to explore and use the system with patients. Nurses across all units felt they missed important training or that training was not long enough. Due to training scheduled during work hours, many said it was difficult to make time in the day to attend the training sessions. Several of the nurses interviewed reported they did not know the full capabilities of the system. Moreover, nurses reported they rarely trained their patients about the features of the system. In addition to training on the use of the iPads, the television-based units included a device that was also a call light and bed control system. Nurses trained the patients on the use of call lights and bed controls for safety. However, nurses did not consistently train patients on the navigation to the various components of the iPET system. The nurses stated the reason navigation training was overlooked was due to the lack of training themselves or a poor understanding of the system. Most nurses learned how to navigate the system from tips shared from their peers on the unit. Based on the recommendations discovered during the interviews with the nurse managers and nursing team leads, the hospitals will develop a more formal training program for the iPET system.

## Discussion

Overall, the nurses perceived that iPET system could enhance patient engagement and positively affect their clinical practice. Hospitals can use iPET for distraction and anxiety reduction, patient education, and augmenting/enhancing several aspects of clinical nursing practice [27].

### Enhanced Training

Advances in health care technology are common. New technologies are usually outdated by the time implementation has occurred. Nurses must learn how to incorporate new technologies into their clinical practice to optimize patient engagement [28]. Comprehensive in-service training might be considered by some as cost prohibitive, but without the proper preparation the nurses would not be exposed to the full capabilities of the iPET system.

On a larger scale, organizations considering implementing an iPET system must show full support in all aspects of implementation and postimplementation. These systems should not be seen as optional tools, but rather just as integral to their practice as the stethoscope. Hospitals must provide sufficient training for nurses on the new system. Time should be built in to allow the nurses to explore all functionalities of the system, including viewing and critiquing any patient education videos that will be available. Moreover, training should be specific to how nurses can use the technology to enhance their practice. Training should include how nurses can use this technology to interact with their patients; iPET distraction features such as music or white noise were shown in our study to calm patients down who were anxious or agitated. This interaction between the nurse and the patient in using the iPET system is imperative especially for use in patient education. Patient discharge education should be introduced at the beginning of their stay and nurses could assess the level of comprehension of education throughout their stay, allowing for opportunities to address issues and identify appropriate resources.

iPET training should be included with every new nurse orientation so that nurses are aware that this is part of their toolkit to use with patients. Nurses are at the forefront of every quality improvement measure and have been tasked with introducing these systems to their patients. If nurses are not well trained in utilizing these systems, or unaware of the benefits that they bring to their patients, there is the possibility that the system will never be used to its full potential.

### Enhancing Nursing Clinical Practice

These systems have the potential to be used as an enhancement to clinical practice. Ongoing communication during the first couple of months postimplementation, including tips to share with their colleagues and training on the new system, is essential in ensuring that nurses are utilizing iPET to its full capacity. Nurses need to be able to share the ways they are using iPET with their patients. For example, several nurses in the study reported using features such as movies and or music as distraction with their anxious patients, which led to a decrease in amount of call lights and requested pain medication. Additionally the quality of patient education could be improved. If patients and their families could view information on certain diseases, new medication, or discharge instructions first through iPET, then the dialog that occurs between physicians and nurses after may be enhanced and would allow more for a collaborative discussion.

### Increasing Patient Engagement

The adoption, use, and development of a strategy for the patients to remain engaged when their care is transitioned to the community are essential. For example, a patient may be more apt to use a personal health record/patient portal at home if they can become comfortable with these systems in the acute care setting. iPET systems could allow patients to choose appropriate nutritional options for their meals, allowing them to feel empowered to make their own decisions. These iPET systems could inform patients about their anticipated treatment plan, including new medications and diagnostic tests, while in the hospital. Patients report that they are unsure about their treatment plan [29]; the use of the iPET system could function in the same way by providing them a roadmap of what to expect while in the hospital.

### Limitations

We recognize several limitations of this research. First, the implementation of all the features of both the television- and tablet-based systems differed across the institutions and units. In some units, not all modules were included in the implementation, and that may have influenced the perceptions of some of the nurses. Our study also looked at both the television- and tablet-based systems; again, they are very different ways to deliver the technology. Televisions in patient’s room are common and expected. The tablet computers were novel technology, and the nursing staff was still getting used to the methods to administer and monitor their use. An additional limitation is that we studied nursing perception of these systems only. We suggest future studies to include patient and caregiver perception of the effectiveness of these systems. Lastly, as mentioned earlier in this paper, there were methodological challenges across the study. Our team worked diligently to mitigate these challenges and deliver the highest-quality data and analysis that was possible.

### Conclusion

The iPET systems described in this study are just one form of the technology used to engage the acute care or inpatient health consumer. Further research will be necessary to determine the best use of these systems in the inpatient setting, especially from a patient perspective, because most of the research has been conducted in the outpatient setting [2]. At the time of this manuscript, separate research into tablet-delivered patient portals in the inpatient setting is in process and should add to this scant body of current knowledge [30]. Tablet, television-based, and other iPET systems have potential to engage patients and family members when properly implemented and incorporated into nurses’ clinical practice [27].

## Abbreviations

ED: emergency department
FREE: focused rapid ethnographic evaluation
HIT: health information technology
IOM: Institute of Medicine
iPET: interactive patient engagement technologies
IT: information technology
TAM: technology acceptance model
